# Post-stroke mortality in ICU patients with serum glucose-potassium ratio: an analysis of MIMIC-IV database

**DOI:** 10.3389/fneur.2025.1578268

**Published:** 2025-04-16

**Authors:** Zhen Yuan, Aoli Chen, Yunqing Zeng, Jiwei Cheng

**Affiliations:** ^1^Department of Neurology, Putuo Hospital, Shanghai University of Traditional Chinese Medicine, Shanghai, China; ^2^Department of Cardiology, Putuo Hospital, Shanghai University of Traditional Chinese Medicine, Shanghai, China

**Keywords:** MIMIC-IV, AIS, glucose, serum potassium, prognosis

## Abstract

**Introduction:**

Acute ischemic stroke (AIS) patients admitted to the intensive care unit (ICU) have a high mortality rate, necessitating the early identification of those at risk of a poor prognosis. This study investigated the association between the blood glucose-to-potassium ratio (GPR) and the prognosis of AIS patients.

**Methods:**

We conducted a retrospective cohort study using data from the Medical Information Mart for Intensive Care IV (MIMIC-IV) database. The primary outcomes were 28-day, 90-day, and 1-year mortality rates following ICU admission. Multivariate Cox proportional hazards regression models were used to estimate adjusted hazard ratios (HRs) with 95% confidence intervals (CIs). Subgroup analyses, Kaplan–Meier survival curves, and restricted cubic spline models were employed to further evaluate the relationship between the GPR and mortality in AIS patients.

**Results:**

A total of 2,636 AIS patients were included in the study, with a mean age of 69.4 ± 15.6 years. The 1-year mortality rate was 36.8% (*n* = 969). After adjusting for confounders, compared with the first quartile (Q1, GPR ≤ 1.39), the 1-year mortality risks for the second quartile (Q2, 1.39 < GPR ≤ 1.74), third quartile (Q3, 1.74 < GPR ≤ 2.25), and fourth quartile (Q4, GPR ≥ 2.25) were HR = 1.17 (95% CI: 0.95–1.43, *p* = 0.132), HR = 1.42 (95% CI: 1.17–1.73, *p* < 0.001), and HR = 1.61 (95% CI: 1.33–1.96, *p* < 0.001), respectively. Similar trends were observed for 28-day and 90-day mortality. Kaplan–Meier (KM) analysis revealed that groups with higher GPRs had higher mortality rates at 28 days, 90 days, and 1 year. Non-linear analysis further confirmed the presence of an inflection point in the association between the GPR and 365-day mortality, which was identified at GPR = 2.75. At ratios less than this threshold, the risk of mortality increased significantly with increasing GPR (HR: 1.466; 95% CI: 1.239–1.735; *p* < 0.001). However, above this ratio, the association plateaued and was no longer statistically significant (HR: 0.899; 95% CI: 0.726–1.113; *p* = 0.095).

**Conclusion:**

The GPR is an independent predictor of poor prognosis in AIS patients admitted to the ICU. Higher GPRs are associated with increased 28-day and 90-day mortality rates, highlighting the potential utility of this ratio in risk stratification and clinical decision-making. A non-linear relationship was observed between the GPR and 365-day mortality, with an inflection point identified at GPR = 2.75.

## 1 Introduction

Stroke is a life-threatening and prevalent condition and is the second leading cause of death worldwide (1). Patients with severe stroke often experience systemic organ failure, which presents significant challenges for subsequent treatment (2). Early identification of key prognostic factors and timely intervention are crucial for improving patient outcomes. However, there is currently no universally recognized objective indicator for predicting the prognosis of patients with severe strokes. Research has shown that during the acute phase of stroke, blood glucose levels can significantly increase (3). In patients with severe acute stroke, dysregulated glucose metabolism can lead to infarct expansion and impaired neurological recovery (4). Additionally, blood potassium levels have been shown to influence the prognosis of stroke. A study involving 421 stroke patients revealed that lower plasma potassium levels were associated with worse clinical outcomes. This may be due to hypokalemia impairing brain cell function, exacerbating ischemic injury, and increasing the risk of arrhythmia (5). Therefore, the coexistence of hyperglycemia and hypokalemia is associated with a poorer stroke prognosis. The ratio of serum glucose to potassium (GPR) has been explored in clinical practice. Previous studies have identified the GPR as a risk factor for aneurysmal subarachnoid hemorrhage (6) and an important biomarker in traumatic brain injury (7). Recent research further suggested that the GPR may serve as a prognostic indicator for intracerebral hemorrhage and coronary artery disease (8, 9).

Previous studies have suggested that the glucose-to-potassium ratio (GPR) may serve as an indicator of the short-term prognosis of ischemic stroke patients (10). However, these studies were limited by small sample sizes and a focus solely on short-term mortality. The impact of the GPR on the long-term prognosis of patients with severe ischemic stroke remains unclear, and no studies have specifically investigated its association with mortality in ICU-admitted acute ischemic stroke (AIS) patients. Therefore, utilizing the MIMIC-IV database, this study included ICU patients with severe AIS to examine the relationship between the GPR and all-cause mortality at 28 days, 90 days, and 1 year in critically ill AIS patients. Our goal was to evaluate whether the GPR could serve as a biomarker for predicting the prognosis of severe AIS patients.

## 2 Materials and methods

### 2.1 Data source

This study is a retrospective cohort study based on the MIMIC-IV 2.2 database. The data were sourced from the Medical Information Mart for Intensive Care IV (MIMIC-IV) 2.2 database, which was developed by the MIT Laboratory for Computational Physiology. The MIMIC-IV database is a publicly available clinical database containing records of 431,231 hospitalized patients at Beth Israel Deaconess Medical Center from 2008–2019. The author of this study, Yuan Zhen, participated in a training program provided by the National Institutes of Health (NIH) and successfully passed the “Protecting Human Research Participants” exam (ID: 65422903). To ensure patient privacy, all personally identifiable information in the database has been encrypted. Therefore, this study does not require ethical approval.

### 2.2 Study population

**Figure 1:**
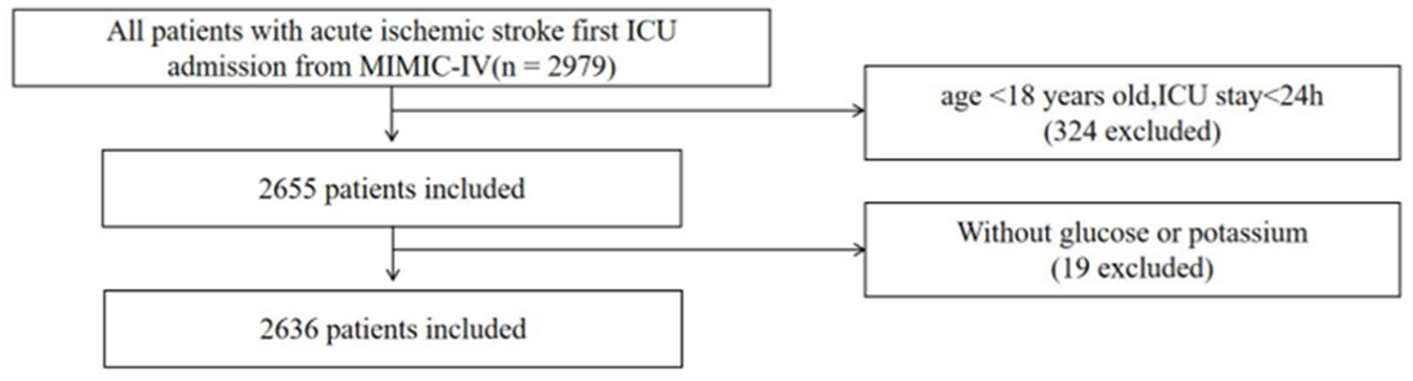
The flow of the enrolled patients throughout the study.

In this study, we identified patients with AIS on the basis of the International Classification of Diseases, Ninth and Tenth Editions (ICD-9 and ICD-10) codes in the MIMIC-IV database. For ICD-9, the included codes were 43300, 43301, 43310, 43311, 43320, 43321, 43330, 43331, 43380, 43381, 43390, 43391, 43400, 43401, 43410, 43411, 43490, 43491, 436, 4370, and 4371. For ICD-10, the included codes were from the I63 series. For patients with multiple ICU admissions, only data from their first ICU stay were included. To ensure the reliability of our findings, we applied strict exclusion criteria: (1) age under 18 years, (2) ICU stay of <24 h, and (3) missing blood glucose or serum potassium test results. Ultimately, 2,636 ICU patients with ischemic stroke met the inclusion criteria. The detailed patient selection process is illustrated in Figure 1. Because the proportion of missing values for all variables was <7%, we used median imputation to handle the missing data.

### 2.3 Study variables

The primary outcome variables were all-cause mortality at 28 days, 90 days, and 1 year, with time defined as the duration from ICU admission to death.

We collected demographic and clinical data, including age, sex, and ethnicity, from ICU-admitted patients. Vital signs included heart rate, systolic blood pressure, diastolic blood pressure, saturation of peripheral oxygen, and respiratory rate. The laboratory parameters included serum sodium, blood urea nitrogen, bicarbonate, creatinine, chloride, the international normalized ratio (INR), the white blood cell count, the platelet count, and hemoglobin. For patients with multiple measurements during ICU admission, the first recorded value was used.

Additionally, we incorporated clinical scores such as the Sequential Organ Failure Assessment (SOFA) score, Charlson Comorbidity Index (CCI), Acute Physiology Score III (APSIII), and Glasgow Coma Scale (GCS), using the mean values recorded during the ICU stay. The comorbidities included myocardial infarction, congestive heart failure, peripheral vascular disease, chronic pulmonary disease, liver disease, diabetes, malignant cancer, sepsis, and hyperlipidemia.

The interventions considered in this study included ventilator use, vasopressor use, renal replacement therapy, mechanical thrombectomy, thrombolysis, potassium supplementation, anticoagulation, and glucose-lowering therapy.

Data extraction was performed using PostgreSQL (version 13.9) and Navicat Premium (version 17.0.4) with Structured Query Language (SQL). All codes used for extracting demographic data, laboratory parameters, comorbidities, and clinical scores were sourced from the GitHub repository: https://github.com/MIT-LCP/MIMIC-code.

### 2.4 Statistical analysis

To increase the robustness of our results, better reflect the data distribution, and facilitate subsequent survival analysis, we referred to established methods in the literature (11) and considered the distribution of GPR data in this study. We categorized the 2,636 patients into quartiles: Q1 (GPR ≤ 1.39), Q2 (1.39 < GPR ≤ 1.74), Q3 (1.74 < GPR ≤ 2.25), and Q4 (GPR ≥ 2.25). Continuous variables, such as age and systolic blood pressure, are expressed as the means ± standard deviations or medians with interquartile ranges, whereas categorical variables, such as sex and disease status, are reported as percentages of outcome events. To compare baseline characteristics among different groups, we applied appropriate statistical tests. For continuous variables, we used ANOVA and the rank-sum test; for categorical variables, we applied the chi-square test and Fisher's exact test. These methods were employed to assess differences in baseline characteristics across the four GPR quartile groups.

We performed Cox univariate regression analysis to evaluate the associations between the GPR and all-cause mortality at 28 days, 90 days, and 1 year. Variables with statistical significance or clinical relevance were selected for inclusion in the Cox multivariate regression models. To ensure the stability of our results, we established three models. Model I was adjusted for sex and age. Model II was further adjusted for race, heart rate, systolic blood pressure, diastolic blood pressure, respiratory rate, serum sodium, blood urea nitrogen, bicarbonate, creatinine, INR, white blood cell count, and hemoglobin. Model III included additional adjustments for the SOFA score, the GCS score, myocardial infarction, congestive heart failure, chronic pulmonary disease, liver disease, diabetes, malignant cancer, vasopressor use, ventilator use, renal replacement therapy, mechanical thrombectomy, thrombolysis, potassium supplementation, anticoagulation, and glucose-lowering therapy.

We assessed survival across the four GPR quartiles via Kaplan–Meier (KM) curves. To account for potential extreme values, we excluded the top and bottom 1% of values before applying a restricted cubic spline regression to model the relationship between GPR and survival at 28 days, 90 days, and 1 year. For non-linear associations, we employed a two-piece Cox proportional hazards model to identify the inflection point and performed separate Cox regression analyses on either side of this threshold. To further validate the robustness of our findings, we conducted subgroup analyses and interaction tests.

All the statistical analyses were performed using R software (version 4.2.0) and Free Statistics software (version 1.8) (12). A two-tailed *p* value < 0.05 was considered statistically significant.

## 3 Results

### 3.1 Baseline characteristics

We identified 2,979 patients who were diagnosed with ischemic stroke upon ICU admission. After applying the inclusion and exclusion criteria, 2,636 patients were included in the study. No patients were lost due to lack of follow-up data in the database. The average age was 69.4 ± 15.6 years, and 1,305 (49.5%) were male. The all-cause mortality rates were 23.8% (*n* = 628) at 28 days, 30.1% (*n* = 794) at 90 days, and 36.8% (*n* = 969) at 1 year. Compared with those in the lowest quartile, a lower proportion of patients in the highest GPR quartile were white individuals (55.8% vs. 64.8%, *P* = 0.006) and were less likely to undergo mechanical thrombectomy (4.4% vs. 5.6%, *P* = 0.007). However, presented significantly greater values for heart rate (88.7 ± 19.9 vs. 81.7 ± 19.9 vs. 81.6 ± 18.1 bpm, *P* = 0.006), systolic blood pressure (138.7 ± 28.6 vs. 134.3 ± 25.9 mmHg, *P* = 0.007), respiratory rate (20.7 ± 6.1 vs. 18.7 ± 5.1 bpm, *P* < 0.001), blood urea nitrogen (19.0 [13.0, 30.0] vs. 18.0 [12.8, 26.0] mg/dL, *P* < 0.001), white blood cell count (11.8 [8.8, 15.9] vs. 8.9 [7.0, 11.8] k/μL, *P* < 0.001), SOFA score (3.0 [1.7, 5.1] vs. 2.2 [1.0, 4.1], *P* < 0.001), Charlson Comorbidity Index (6.9 ± 2.9 vs. 6.5 ± 3.0, *P* < 0.001), and APSIII score (45.0 [33.0, 60.0] vs. 37.0 [26.0, 48.0], *P* < 0.001).

Additionally, patients in the highest GPR quartile were more likely to have myocardial infarction (22.2% vs. 15.7%, *P* < 0.001), congestive heart failure (28.8% vs. 26.5%, *P* = 0.040), diabetes (62.5% vs. 15.9%, *P* < 0.001), and sepsis (59.5% vs. 36.6%, *P* < 0.001). They were also more likely to receive vasopressors (38.8% vs. 26.4%, *P* < 0.001), mechanical ventilation (82.2% vs. 61.4%, *P* < 0.001), potassium supplementation (79.1% vs. 54.2%, *P* < 0.001), and glucose-lowering therapy (86.2% vs. 60.8%, *P* < 0.001). The detailed baseline characteristics are presented in Table 1.

**Table 1:** Baseline characteristics of study population.

**Variables**	**Total (*n =* 2,636)**	**Baseline serum glucose potassium ratio levels**				***P-*value**
		**Q1**	**Q2**	**Q3**	**Q4**	
		≤**1.39**	**1.39–1.74**	**1.74–2.25**	**>2.25**	
		**(*****n** =* **648)**	**(*****n** =* **668)**	**(*****n** =* **661)**	**(*****n** =* **659)**	
Age (year)	69.4 ± 15.6	69.1 ± 16.9	68.6 ± 16.1	70.6 ± 14.9	69.3 ± 14.4	0.105
Male (%)	1,305 (49.5)	334 (51.5)	352 (52.7)	311 (47)	308 (46.7)	0.06
Ethnicity, white (%)	1,615 (61.3)	420 (64.8)	421 (63)	406 (61.4)	368 (55.8)	0.006
**Vital signs**						
Heart rate (bpm)	84.2 ± 19.0	81.6 ± 18.1	81.9 ± 18.1	84.7 ± 19.1	88.7 ± 19.9	<0.001
SBP (mmHg)	137.5 ± 26.9	134.3 ± 25.9	138.6 ± 25.6	138.2 ± 27.0	138.7 ± 28.6	0.007
DBP (mmHg)	74.7 ± 18.8	73.8 ± 17.9	75.9 ± 18.5	74.6 ± 19.2	74.5 ± 19.5	0.237
SPO_2_ (%)	97.2 ± 3.6	97.3 ± 3.3	97.4 ± 3.8	97.5 ± 3.0	96.9 ± 4.1	0.025
Resp rate (bpm)	19.2 ± 5.5	18.7 ± 5.1	18.4 ± 5.3	18.9 ± 5.3	20.7 ± 6.1	<0.001
**Laboratory results**						
Sodium (mmol/L)	139.3 ± 4.7	139.3 ± 4.1	139.6 ± 4.3	139.3 ± 4.7	138.9 ± 5.5	0.062
BUN (mg/dL)	17.0 (13.0, 26.0)	18.0 (12.8, 26.0)	16.0 (12.0, 23.0)	18.0 (13.0, 25.0)	19.0 (13.0, 30.0)	<0.001
Bicarbonate (mEq/L)	22.9 ± 3.9	22.6 ± 3.8	23.1 ± 3.3	23.5 ± 3.6	22.2 ± 4.4	<0.001
Scr (mg/dL)	0.9 (0.7, 1.3)	0.9 (0.8, 1.4)	0.9 (0.7, 1.2)	0.9 (0.7, 1.2)	1.0 (0.8, 1.4)	<0.001
Chloride (mEq/L)	104.3 ± 5.8	104.7 ± 5.2	104.9 ± 5.3	104.2 ± 5.6	103.4 ± 6.7	<0.001
INR	1.2 (1.1, 1.3)	1.2 (1.1, 1.4)	1.2 (1.1, 1.3)	1.2 (1.1, 1.4)	1.2 (1.1, 1.3)	0.075
WBC (k/uL)	10.2 (7.7, 13.7)	8.9 (7.0, 11.8)	9.7 (7.6, 13.1)	10.6 (8.1, 13.9)	11.8 (8.8, 15.9)	<0.001
Platelets (k/uL)	206.0 (157.0, 263.0)	202.5 (151.8, 257.0)	203.5 (160.0, 255.2)	207.0 (158.0, 264.0)	211.0 (157.5, 275.5)	0.333
Hemoglobin (g/dL)	11.5 ± 2.3	11.3 ± 2.4	11.7 ± 2.2	11.6 ± 2.3	11.5 ± 2.4	0.01
Potassium (mmol/L)	4.1 ± 0.7	4.5 ± 0.7	4.1 ± 0.5	4.0 ± 0.6	3.9 ± 0.7	<0.001
Glucose (mmol/L)	8.0 ± 4.1	5.3 ± 0.8	6.4 ± 0.9	7.8 ± 1.3	12.6 ± 5.6	<0.001
**Score system, points**						
SOFA score	2.5 (1.0, 4.3)	2.2 (1.0, 4.1)	2.0 (1.0, 3.7)	2.6 (1.3, 4.2)	3.0 (1.7, 5.1)	<0.001
CCI	6.5 ± 2.9	6.5 ± 3.0	6.0 ± 2.7	6.5 ± 2.8	6.9 ± 2.9	<0.001
APSIII	38.0 (28.0, 52.0)	37.0 (26.0, 49.0)	34.0 (26.0, 48.0)	37.0 (29.0, 51.0)	45.0 (33.0, 60.0)	<0.001
GCS	13.9 ± 1.7	14.1 ± 1.4	14.0 ± 1.6	13.8 ± 1.9	13.8 ± 1.8	0.002
**Comorbidity disease**						
Myocardial infarct (%)	441 (16.7)	102 (15.7)	88 (13.2)	105 (15.9)	146 (22.2)	<0.001
Congestive heart failure (%)	689 (26.1)	172 (26.5)	148 (22.2)	179 (27.1)	190 (28.8)	0.04
Peripheral vascular disease (%)	353 (13.4)	84 (13)	96 (14.4)	83 (12.6)	90 (13.7)	0.779
Chronic pulmonary disease (%)	507 (19.2)	133 (20.5)	113 (16.9)	117 (17.7)	144 (21.9)	0.075
Liver disease (%)	165 (6.3)	36 (5.6)	31 (4.6)	47 (7.1)	51 (7.7)	0.078
Diabetes (%)	863 (32.7)	103 (15.9)	125 (18.7)	223 (33.7)	412 (62.5)	<0.001
Malignant cancer (%)	229 (8.7)	58 (9)	51 (7.6)	58 (8.8)	62 (9.4)	0.699
Sepsis (%)	1,201 (45.6)	237 (36.6)	263 (39.4)	309 (46.7)	392 (59.5)	<0.001
Hyperlipidemia (%)	1,187 (45.0)	290 (44.8)	292 (43.7)	300 (45.4)	305 (46.3)	0.816
**Interventions**						
Ventilator use (%)	1,925 (73.0)	398 (61.4)	472 (70.7)	513 (77.6)	542 (82.2)	<0.001
Vasopressor use (%)	824 (31.3)	171 (26.4)	182 (27.2)	215 (32.5)	256 (38.8)	<0.001
Mechanical thrombectomy (%)	170 (6.4)	36 (5.6)	59 (8.8)	46 (7)	29 (4.4)	0.007
Thrombolysis (%)	290 (11.0)	68 (10.5)	82 (12.3)	83 (12.6)	57 (8.6)	0.085
Potassium supplementation (%)	1,832 (69.5)	351 (54.2)	469 (70.2)	491 (74.3)	521 (79.1)	<0.001
Anticoagulation (%)	1,995 (75.7)	478 (73.8)	506 (75.7)	499 (75.5)	512 (77.7)	0.431
Glucose-lowering therapy (%)	1,872 (71.0)	394 (60.8)	439 (65.7)	471 (71.3)	568 (86.2)	<0.001
RRT use (%)	97 (3.7)	33 (5.1)	21 (3.1)	17 (2.6)	26 (3.9)	0.085
28-day mortality (%)	628 (23.8)	122 (18.8)	134 (20.1)	162 (24.5)	210 (31.9)	<0.001
90-day mortality (%)	794 (30.1)	158 (24.4)	171 (25.6)	210 (31.8)	255 (38.7)	<0.001
365-day mortality (%)	969 (36.8)	193 (29.8)	206 (30.8)	262 (39.6)	308 (46.7)	<0.001

SBP, systolic blood pressure; DBP, diastolic blood pressure; Resp, Respiration; SPO_2_, saturation of peripheral oxygen; BUN, blood urea nitrogen; SCr, serum creatinine; INR, international normalized ratio; WBC, white blood count; SOFA, score sequential organ failure assessment score; CCI, charlson comorbidity index; APS III, acute physiology score III; GCS, glasgow Coma Scale; RRT, renal replacement therapy.

### 3.2 Multivariate cox regression analysis

After univariate regression analysis (Supplementary Table S1), three multivariate Cox regression models were established on the basis of the univariate analysis results and clinical considerations to explore the relationship between the GPR and mortality.

In the unadjusted model, when the GPR was treated as a continuous variable, it was significantly associated with 28-day mortality (HR: 1.10; 95% CI: 1.06–1.14; *P* < 0.001), 90-day mortality (HR: 1.09; 95% CI: 1.05–1.13; *P* < 0.001), and 1-year mortality (HR: 1.09; 95% CI: 1.06–1.13; *P* < 0.001). When the GPR was analyzed as a categorical variable, patients in the highest quartile had significantly greater 28-day mortality (HR: 1.87; 95% CI: 1.50–2.34; *P* < 0.001), 90-day mortality (HR: 1.79; 95% CI: 1.47–2.18; *P* < 0.001), and 1-year mortality (HR: 1.81; 95% CI: 1.51–2.16; *P* < 0.001) than did those in the lowest quartile.

After adjusting for sex, age, race, heart rate, systolic blood pressure, diastolic blood pressure, respiratory rate, serum sodium, blood urea nitrogen, bicarbonate, creatinine, INR, white blood cell count, hemoglobin, SOFA score, GCS, myocardial infarction, congestive heart failure, chronic pulmonary disease, liver disease, diabetes, malignant cancer, vasopressor use, ventilator use, renal replacement therapy, mechanical thrombectomy, thrombolysis, potassium supplementation, anticoagulation, and glucose-lowering therapy, we found that the GPR remained an independent predictor of 28-day mortality (HR: 1.08; 95% CI: 1.02–1.15; *P* = 0.009), 90-day mortality (HR: 1.06; 95% CI: 1.01–1.12; *P* = 0.023), and 1-year mortality (HR: 1.07; 95% CI: 1.02–1.12; *P* = 0.008) when treated as a continuous variable. When the GPR was analyzed as a categorical variable, 28-day mortality (HR: 1.60; 95% CI: 1.24–2.05; *P* < 0.001), 90-day mortality (HR: 1.51; 95% CI: 1.21–1.90; *P* < 0.001), and 1-year mortality (HR: 1.58; 95% CI: 1.29–1.93; *P* < 0.001) remained significantly higher in the highest quartile than in the lowest quartile according to Cox multivariate regression analysis, indicating that elevated GPR levels may increase the risk of mortality in patients. Table 2 presents the detailed statistical results.

**Table 2:** Multivariate analysis of risk factors for 28-day mortality, 90-day mortality, and 365-day mortality in acute ischemic stroke.

**Variable**	**Crude model**		**Model I**		**Model II**		**Model III**	
	**HR (95% CI)**	***P*** **-value**	**HR (95% CI)**	***P*** **-value**	**HR (95% CI)**	***P*** **-value**	**HR (95% CI)**	***P*** **-value**
**28-day mortality**								
GPR as continuous	1.1 (1.06~1.14)	<0.001	1.12 (1.08~1.16)	<0.001	1.06 (1~1.12)	0.035	1.08 (1.02~1.15)	0.009
GPR ≤ 1.39	1(Ref)		1(Ref)		1(Ref)		1(Ref)	
1.39 < GPR ≤ 1.74	1.07 (0.84~1.37)	0.594	1.1 (0.86~1.4)	0.462	1.2 (0.93~1.55)	0.151	1.06 (0.83~1.37)	0.626
1.74 < GPR ≤ 2.25	1.36 (1.08~1.72)	0.01	1.36 (1.07~1.72)	0.011	1.38 (1.08~1.76)	0.011	1.23 (0.96~1.57)	0.095
GPR > 2.25	1.87 (1.5~2.34)	<0.001	1.92 (1.54~2.4)	<0.001	1.6 (1.26~2.03)	<0.001	1.6 (1.24~2.05)	<0.001
P for trend		<0.001		<0.001		<0.001		<0.001
**90-day mortality**								
GPR as continuous	1.09 (1.05~1.13)	<0.001	1.11 (1.07~1.15)	<0.001	1.05 (1~1.1)	0.065	1.06 (1.01~1.12)	0.023
GPR ≤ 1.39	1(Ref)		1(Ref)		1(Ref)		1(Ref)	
1.39 < GPR ≤ 1.74	1.06 (0.85~1.31)	0.619	1.08 (0.87~1.35)	0.463	1.17 (0.93~1.46)	0.178	1.07 (0.85~1.33)	0.574
1.74 < GPR ≤ 2.25	1.38 (1.12~1.69)	0.002	1.37 (1.11~1.68)	0.003	1.37 (1.1~1.7)	0.004	1.24 (1~1.53)	0.055
GPR > 2.25	1.79 (1.47~2.18)	<0.001	1.84 (1.5~2.24)	<0.001	1.52 (1.23~1.89)	<0.001	1.51 (1.21~1.9)	<0.001
P for trend		<0.001		<0.001		<0.001		<0.001
**365-day mortality**								
GPR as continuous	1.09 (1.06~1.13)	<0.001	1.11 (1.07~1.15)	<0.001	1.06 (1.01~1.11)	0.011	1.07 (1.02~1.12)	0.008
GPR ≤ 1.39	1(Ref)		1(Ref)		1(Ref)		1(Ref)	
1.39 < GPR ≤ 1.74	1.05 (0.86~1.27)	0.659	1.07 (0.88~1.31)	0.472	1.17 (0.95~1.43)	0.132	1.08 (0.89~1.33)	0.436
1.74 < GPR ≤ 2.25	1.43 (1.19~1.72)	<0.001	1.42 (1.18~1.71)	<0.001	1.42 (1.17~1.73)	<0.001	1.32 (1.08~1.6)	0.006
GPR>2.25	1.81 (1.51~2.16)	<0.001	1.86 (1.56~2.23)	<0.001	1.61 (1.33~1.96)	<0.001	1.58 (1.29~1.93)	<0.001
P for trend		<0.001		<0.001		<0.001		<0.001

Crude model was not adjusted. Model 1 was adjusted for Gender + Age. Model 2 was adjusted for model 1 + Ethnicity + HR + SBP + DBP + Resp + Na^+^ + Bun + ${\text{HCO}}_{3}^{-}$ + Scr + INR + WBC + Hb. Model 3 was adjusted for model 2 + SOFA score + GCS + MI + CHF + COPD + Liver disease + DM + Malignant cancer + MV + VP + RRT + MT + TL + KS + AC + GLT. Na^+^, serum sodium; HCO3^−^, arterial bicarbonate; Hb, Hemoglobin; MI, myocardial infarct; CHF, congestive heart failure; COPD, chronic pulmonary disease; DM, diabetes; MV, mechanical ventilation; VP, vasopressor; MT, mechanical thrombectomy; TL, thrombolysis; KS, potassium supplementation; AC, anticoagulation; GLT, glucose-lowering therapy.

### 3.3 Kaplan–Meier survival curves

**Figure 2:**
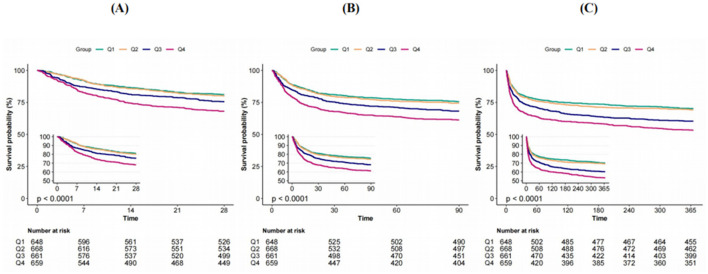
Kaplan-Meier survival analysis curves for **(A)** 28-day, **(B)** 90-day, and **(C)** 1 year ACM.

When the KM survival curves of severe stroke patients divided into four groups based on the quartiles of the GPR were analyzed, the GPR was found to be positively correlated with mortality rates at 28 days, 90 days, and 1 year. The higher the GPR was, the higher the mortality rate was (*P* < 0.0001), and this correlation increased over time. The detailed survival curves are shown in Figure 2.

### 3.4 Analyses of the non-linear relationship

**Figure 3:**
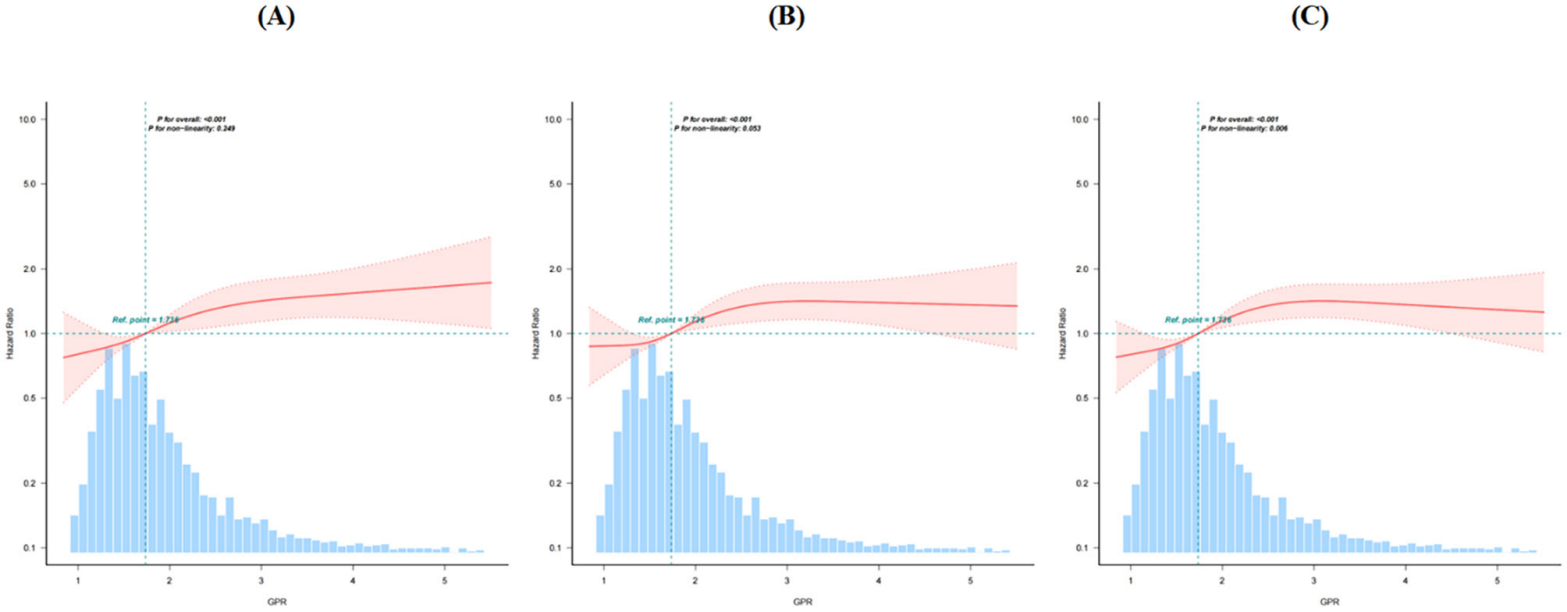
Restricted cubic spline curve for **(A)** 28-day, **(B)** 90-day, and **(C)** 1 year ACM.

By fitting the curve and adjusting for confounding variables, mortality rates at 28 days and 90 days increased linearly with the GPR. However, a non-linear relationship was identified for 1-year mortality (*P* = 0.006), as shown in Figure 3. Using a two-part Cox proportional hazards model with a recursive approach, the inflection point was determined to be GPR = 2.75. Below this threshold, GPR was significantly positively associated with 1-year mortality, with a 46.6% increase in mortality per unit of GPR (HR: 1.466; 95% CI: 1.239–1.735; *P* < 0.001). However, when the GPR exceeded 2.75, the association between the GPR and 1-year mortality was no longer statistically significant (HR: 0.899; 95% CI: 0.726–1.113; *P* = 0.0951). The detailed results are listed in Table 3.

**Table 3:** Threshold effect analysis of the relationship between osmo-lality and 365-day mortality of patients with acute ischemic stroke.

**Threshold of GPR**	**HR (95%CI)**	***P* Value**
<2.75	1.466 (1.239,1.735)	<0.001
≥2.75	0.899 (0.726,1.113)	0.0951
Likelihood Ratio test		0.003

The data were adjusted for all the covariates of Model 3.

### 3.5 Subgroup and sensitivity analyses

**Figure 4:**
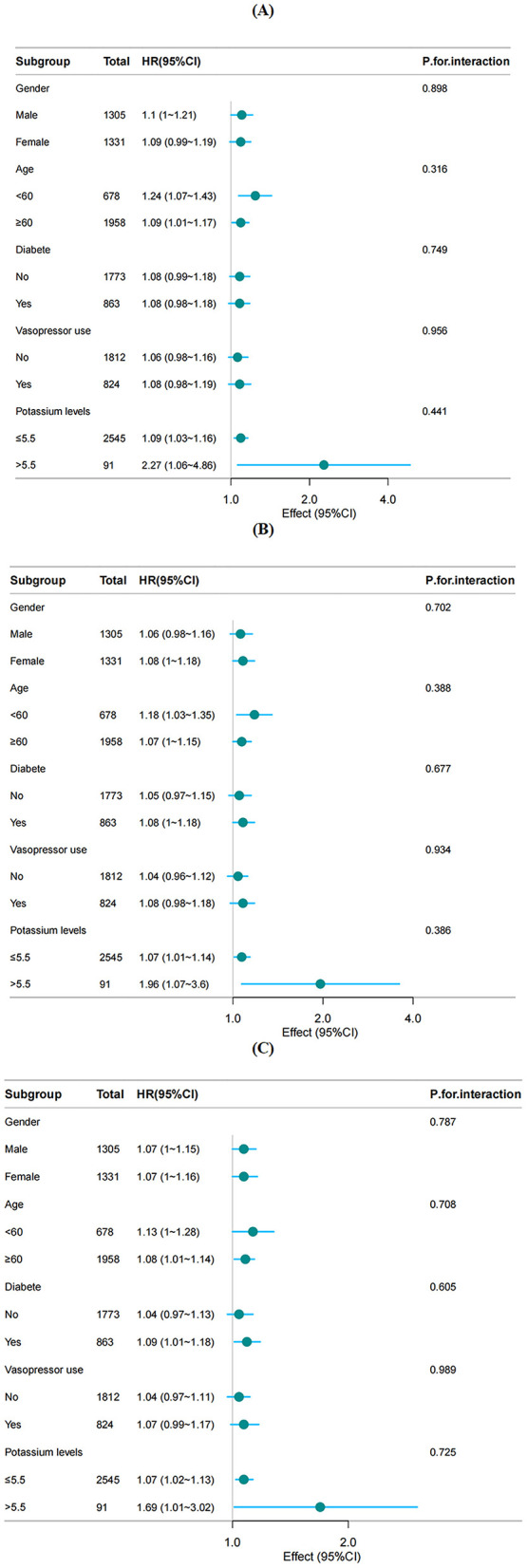
Forest plots of hazard ratios for the **(A)** 28-day, **(B)** 90-day, **(C)** 365-day mortality in different subgroups.

Subgroup analysis confirmed the robustness of the association between the GPR and all-cause mortality in severe AIS patients across different subgroups. In the stratified analysis considering age, sex, diabetes status, vasopressin use, and blood potassium levels, the forest plot (Figure 4) revealed no significant interaction between the GPR and any subgroup (interaction *P* values: 0.259–0.989). These findings further support the GPR as an independent prognostic factor in severe AIS patients.

## 4 Discussion

This study explored the relationship between the glucose-to-potassium ratio (GPR) and the prognosis of ICU-admitted ischemic stroke patients. Multivariate Cox proportional hazards regression analysis identified the baseline GPR as an independent predictor of all-cause mortality at 28 days, 90 days, and 1 year. When the GPR was treated as a continuous variable, a significant positive correlation was observed with 28-day mortality (HR: 1.08; 95% CI: 1.02–1.15; *P* = 0.009). When the GPR was categorized into quartiles, patients in the highest quartile (Q4) had a significantly higher 28-day mortality rate than those in the lowest quartile (HR: 1.6; 95% CI: 1.24–2.05; *P* < 0.001). These findings align with those of a previous single-center study conducted in Norway (10), which included 784 emergency department patients between 2010 and 2015. In that study, when the GPR was modeled as a continuous variable, a significant positive correlation with mortality was observed (OR: 2.01; 95% CI: 1.12–3.61; *P* = 0.01). When the GPR was categorized, the odds ratios (ORs) and 95% confidence intervals (CIs) for the second and third quartiles relative to the first quartile were 1.24 (0.60–2.56) and 2.15 (1.09–4.24), respectively (p for trend = 0.0188). Our study also revealed that higher GPRs were positively associated with 90-day mortality (HR: 1.09; 95% CI: 1.05–1.13; *P* < 0.001). When treated as a categorical variable, patients in the highest GPR quartile (Q4) exhibited significantly increased 90-day mortality (HR: 1.79; 95% CI: 1.47–2.18; *P* < 0.001). This finding aligns with a previous single-center retrospective study conducted in Nanjing, China (13), which included ischemic stroke patients who underwent endovascular thrombectomy (EVT) between 2019 and 2021. That study demonstrated a significant association between elevated GPR and unfavorable 90-day outcomes (OR: 1.852; 95% CI: 1.276–2.688; *P* = 0.001). Another retrospective study from Nanjing involving 1,826 patients with acute ischemic stroke, reported a U-shaped association between the GPR and poor 90-day prognosis (14). In terms of long-term outcomes, our study also revealed that patients in the highest GPR quartile had a significantly increased 1-year mortality risk (HR: 1.58; 95% CI: 1.29–1.93; *P* < 0.001), which is consistent with findings from a multicenter study conducted in Saudi Arabia. That study included 1,047 ischemic stroke patients between 2016 and 2022 and reported significantly higher 12-month all-cause mortality in patients with a GPR ≥ 1.67 than in those with a GPR < 1.67 (aHR: 2.07; 95% CI: 1.21–3.75; *P* = 0.01) (15). Compared with the above studies (10, 13–15), our research included a larger sample size, enhancing the robustness and generalizability of the results. Additionally, previous studies (10, 13–15) did not account for key variables such as renal function, the international normalized ratio (INR), or renal replacement therapy. Our analysis included these indicators, resulting in a more comprehensive evaluation and offering more reliable conclusions with greater clinical relevance. Furthermore, while prior studies (10, 13, 14) predominantly used logistic regression models, they did not account for the time-dependent nature of mortality. In contrast, we employed a more rigorous Cox proportional hazards model, which fully incorporated time-to-event data and improved the validity of our findings. Moreover, an elevated GPR has also been associated with poor outcomes in patients with severe traumatic brain injury, aneurysmal subarachnoid hemorrhage, and hemorrhagic moyamoya disease (16–18). To date, however, no high-quality studies have specifically examined the GPR as a prognostic marker in critically ill patients with ischemic stroke admitted to the ICU. Therefore, the aim of this study is to close that important research gap.

The underlying mechanisms by which the GPR serves as a prognostic indicator for ischemic stroke remain incompletely understood. However, existing studies suggest that both hyperglycemia and hypokalemia play significant roles in stroke pathophysiology. Ischemic stroke, a severe traumatic and stress-inducing event, can activate the hypothalamic–pituitary–adrenal (HPA) axis, leading to elevated serum glucocorticoid levels and activation of the sympathetic autonomic nervous system (SANS), which triggers the release of catecholamines (19, 20). Catecholamines enhance aerobic glycolysis, glycogen breakdown, and gluconeogenesis while inhibiting insulin-mediated glycogen synthesis, ultimately leading to hyperglycemia (20). Even in non-diabetic patients, this response can induce stress-induced transient hyperglycemia (21). Elevated blood glucose increases vascular viscosity and promotes inflammatory responses, generating oxygen free radicals that contribute to diffuse small-vessel disease, exacerbating brain tissue ischemia and edema (22). Clinical studies have further confirmed that hyperglycemia is associated with an increased risk of stroke recurrence (23). Potassium levels also have a significant effect on stroke prognosis. Stroke can cause excessive secretion of catecholamines and insulin, which modulate the transmembrane transport of potassium via the Na+/K+-ATPase pump, leading to intracellular potassium influx and a subsequent decrease in serum potassium levels (24). Additionally, stroke can induce aldosterone hypersecretion, which overactivates the renin–angiotensin–aldosterone system (RAAS), accelerating renal potassium excretion and causing hypokalemia (25). Hypokalemia can impair both vascular smooth muscle cells and vasoconstrictor receptors, further compromising cerebral perfusion (5). Moreover, studies have shown that low potassium levels are a risk factor for ischemic stroke recurrence (26), whereas increased dietary potassium intake has a protective effect against stroke (27, 28). In conclusion, there is a complex interplay between serum glucose, blood potassium, and ischemic stroke. As a readily measurable emergency biomarker, the GPR provides valuable prognostic information and can serve as a useful tool for predicting the outcomes of ischemic stroke in clinical practice.

Interestingly, higher GPRs were associated with a lower rate of mechanical thrombectomy. This may be explained by the potential of thrombectomy to improve cerebral infarction outcomes, reduce stress responses, and consequently lower blood glucose levels. However, the precise mechanisms underlying this association remain to be elucidated. Another notable finding emerged during the curve fitting analysis. We observed a linear relationship between the GPR and 28-day and 90-day mortality rates. However, for 1-year mortality, an inverted L-shaped non-linear relationship emerged, with a turning point at GPR = 2.75. When the GPR was < 2.75, a positive correlation with mortality remained, indicating that as the GPR increased, the risk of death continued to rise. However, when the GPR was ≥ 2.75, the correlation between the GPR and mortality plateaued, suggesting a saturation effect (29). From a clinical perspective, when the GPR is < 2.75 and continues to rise, persistent hyperglycemia or hypokalemia is possible, both of which are modifiable risk factors. In such cases, enhanced blood glucose control (e.g., insulin therapy) and potassium management (e.g., potassium supplementation) should be considered to mitigate mortality risk. However, when the GPR is > 2.75, mortality is likely influenced by additional factors such as organ failure and infection, meaning that adjusting glucose and potassium alone may not significantly affect patient prognosis. These findings emphasize the need for early individualized treatment strategies based on GPR levels to prevent further increases in this level. In clinical practice, hyperkalemia is often associated with increased mortality but a decreased GPR. To further investigate this association, we performed a subgroup analysis of hyperkalaemic patients, which confirmed that even within this population, the GPR remained positively correlated with mortality.

This study has several limitations. First, we measured only the initial GPR upon ICU admission, without monitoring its dynamic changes during the ICU stay, which may have provided additional insights. Second, owing to database constraints, we were unable to obtain key hormonal data (e.g., insulin, corticosteroids, glucagon, and catecholamines), all of which significantly influence glucose metabolism. Moreover, the unavailability of imaging data limits our ability to stratify patients by stroke etiology (e.g., lacunar vs. embolic stroke), thereby limiting further mechanistic insights. The absence of National Institutes of Health Stroke Scale (NIHSS) scores may also confound the association between the GPR and mortality, as higher GPR values may be correlated with greater stroke severity. Similarly, the lack of detailed treatment-related information restricts the comprehensiveness and generalizability of our findings. Finally, as a single-center, retrospective study, our results should be interpreted with caution, and further large-scale, multicenter prospective studies are needed to validate and expand upon our findings.

## 5 Conclusion

The GPR is an independent predictor of poor prognosis in AIS patients admitted to the ICU. Higher GPR is associated with increased 28-day and 90-day mortality rates, highlighting its potential utility in risk stratification and clinical decision-making. A non-linear relationship was observed between the GPR and 365-day mortality, with an inflection point identified at GPR = 2.75.

## Data Availability

The datasets presented in this study can be found in online repositories. The names of the repository/repositories and accession number(s) can be found below: https://physionet.org/, doi: 10.13026/6mm1-ek67.
