# Supplementary material for: Post-stroke mortality in ICU patients with serum glucose-potassium ratio: an analysis of MIMIC-IV database
Source: Front Neurol. 2025 Apr 16;16:1578268. doi: 10.3389/fneur.2025.1578268 (PMC12040655; doi:10.3389/fneur.2025.1578268)
Supplement: Supplementary file 1 [file Table_1.docx]

**TABLE S1** | Univariate analysis of risk factors for 28-day mortality,90-day mortality and 365-day mortality in acute ischemic stroke.

| Characteristic | **28-Day Mortality** | | **90-Day Mortality** | | **365-Day Mortality** | |
| --- | --- | --- | --- | --- | --- | --- |
|  | HR(95%CI) | P value | HR(95%CI) | P value | HR(95%CI) | P value |
| Age | 1.03 (1.02,1.03) | < 0.001 | 1.03 (1.02,1.03) | < 0.001 | 1.03 (1.03,1.03) | < 0.001 |
| Gender | 0.87 (0.75,1.02) | 0.094 | 0.87 (0.76,1) | 0.057 | 0.88 (0.78,1) | 0.05 |
| Ethnicity | 0.77 (0.66,0.9) | 0.001 | 0.8 (0.7,0.92) | 0.002 | 0.85 (0.75,0.97) | 0.015 |
| Heart rate | 1.01 (1.01,1.02) | < 0.001 | 1.01 (1.01,1.02) | < 0.001 | 1.01 (1.01,1.01) | < 0.001 |
| SBP | 0.9964 (0.9934,0.9994) | 0.018 | 0.9958 (0.9932,0.9985) | 0.002 | 0.9971 (0.9947,0.9995) | 0.019 |
| DBP | 0.9965 (0.9922,1.0008) | 0.109 | 0.996 (0.9921,0.9998) | 0.038 | 0.9956 (0.9921,0.9991) | 0.013 |
| SPO2 | 0.9912 (0.9709,1.012) | 0.416 | 0.99 (0.97,1.01) | 0.176 | 0.99 (0.97,1.01) | 0.226 |
| Resp rate | 1.04 (1.02,1.05) | < 0.001 | 1.04 (1.03,1.05) | < 0.001 | 1.04 (1.02,1.05) | < 0.001 |
| Sodium | 1.02 (1,1.04) | 0.034 | 1.01 (1,1.03) | 0.12 | 1.01 (1,1.02) | 0.151 |
| Bun | 1.01 (1.01,1.02) | < 0.001 | 1.02 (1.01,1.02) | < 0.001 | 1.02 (1.01,1.02) | < 0.001 |
| Bicarbonate | 0.94 (0.92,0.95) | < 0.001 | 0.95 (0.93,0.96) | < 0.001 | 0.96 (0.94,0.97) | < 0.001 |
| Scr | 1.05 (1.02,1.09) | 0.01 | 1.06 (1.03,1.09) | < 0.001 | 1.07 (1.05,1.1) | < 0.001 |
| Chloride | 1.006 (0.9922,1.0199) | 0.395 | 0.9986 (0.9866,1.0109) | 0.827 | 0.9954 (0.9845,1.0065) | 0.416 |
| INR | 1.21 (1.11,1.31) | < 0.001 | 1.21 (1.13,1.3) | < 0.001 | 1.22 (1.15,1.3) | < 0.001 |
| WBC | 1.01 (1.01,1.02) | < 0.001 | 1.01 (1.01,1.01) | < 0.001 | 1.01 (1.01,1.01) | < 0.001 |
| Platelets | 1 (0.9992,1.0008) | 0.97 | 0.9997 (0.999,1.0004) | 0.426 | 0.9996 (0.999,1.0003) | 0.258 |
| Hemoglobin | 0.91 (0.88,0.94) | < 0.001 | 0.9 (0.87,0.93) | < 0.001 | 0.89 (0.87,0.92) | < 0.001 |
| SOFA score | 1.14 (1.11,1.16) | < 0.001 | 1.15 (1.12,1.17) | < 0.001 | 1.14 (1.12,1.16) | < 0.001 |
| CCI | 1.16 (1.13,1.19) | < 0.001 | 1.18 (1.16,1.21) | < 0.001 | 1.2 (1.17,1.22) | < 0.001 |
| APSIII | 1.02 (1.02,1.03) | < 0.001 | 1.02 (1.02,1.03) | < 0.001 | 1.02 (1.02,1.03) | < 0.001 |
| GCS | 0.86 (0.83,0.89) | < 0.001 | 0.86 (0.83,0.89) | < 0.001 | 0.86 (0.84,0.89) | < 0.001 |
| Myocardial infarct | 1.43 (1.18,1.72) | < 0.001 | 1.48 (1.25,1.75) | < 0.001 | 1.51 (1.29,1.76) | < 0.001 |
| Congestive heart failure | 1.45 (1.23,1.71) | < 0.001 | 1.63 (1.41,1.88) | < 0.001 | 1.69 (1.48,1.92) | < 0.001 |
| Peripheral vascular disease | 1.06 (0.84,1.32) | 0.638 | 1.03 (0.84,1.26) | 0.751 | 1.08 (0.9,1.3) | 0.389 |
| Chronic pulmonary disease | 1.22 (1.01,1.47) | 0.044 | 1.25 (1.06,1.48) | 0.009 | 1.31 (1.13,1.52) | < 0.001 |
| Liver disease | 1.62 (1.24,2.12) | < 0.001 | 1.72 (1.36,2.17) | < 0.001 | 1.54 (1.23,1.94) | < 0.001 |
| Diabetes | 1.03 (0.87,1.22) | 0.714 | 1.09 (0.95,1.27) | 0.231 | 1.18 (1.04,1.35) | 0.014 |
| Malignant cancer | 2.09 (1.68,2.6) | < 0.001 | 2.3 (1.9,2.79) | < 0.001 | 2.4 (2.02,2.86) | < 0.001 |
| Sepsis | 2.02 (1.72,2.38) | < 0.001 | 2.09 (1.82,2.42) | < 0.001 | 2.07 (1.82,2.36) | < 0.001 |
| Hyperlipidemia | 0.86 (0.74,1.01) | 0.073 | 0.88 (0.77,1.02) | 0.087 | 0.89 (0.78,1.01) | 0.067 |
| Ventilator use | 3.54 (2.74,4.57) | < 0.001 | 3.09 (2.5,3.83) | < 0.001 | 2.75 (2.29,3.3) | < 0.001 |
| Vasopressor use | 1.94 (1.65,2.27) | < 0.001 | 1.95 (1.69,2.24) | < 0.001 | 1.75 (1.54,1.99) | < 0.001 |
| Mechanical thrombectomy | 1.03 (0.76,1.42) | 0.831 | 1.06 (0.8,1.4) | 0.677 | 1.05 (0.81,1.35) | 0.716 |
| Thrombolysis | 0.82 (0.63,1.07) | 0.151 | 0.76 (0.59,0.97) | 0.026 | 0.82 (0.66,1.02) | 0.072 |
| Potassium supplementation | 1.4 (1.17,1.68) | < 0.001 | 1.44 (1.22,1.7) | < 0.001 | 1.37 (1.19,1.59) | < 0.001 |
| Anticoagulation | 0.73 (0.62,0.87) | < 0.001 | 0.79 (0.68,0.93) | 0.003 | 0.83 (0.72,0.95) | 0.009 |
| Glucose-lowering therapy | 1.12 (0.94,1.33) | 0.224 | 1.11 (0.95,1.29) | 0.209 | 1.15 (1,1.33) | 0.052 |
| RRT | 1.62 (1.15,2.28) | 0.01 | 1.98 (1.49,2.63) | < 0.001 | 1.89 (1.45,2.47) | < 0.001 |
